# Challenges in the Monitoring of Therapeutic Plasma Exchange during Acute Heparin-Induced Thrombocytopenia in Adults under ECMO

**DOI:** 10.1055/a-2277-4404

**Published:** 2024-03-28

**Authors:** Nicolas Gendron, Candice Cavalie, Elie Kantor, Sophie Provenchère, Romain Sonneville, Vasiliki Gkalea, Marie-Charlotte Bourrienne, Dorothée Faille, Nadine Ajzenberg

**Affiliations:** 1 Innovative Therapies in Haemostasis, Paris Cité University, INSERM, Paris, France; 2 Hematology Department, Hôpital Européen Georges-Pompidou, Assistance Publique Hôpitaux de Paris, Centre-Université de Paris (APHP-CUP), Paris, France; 3 Université de Paris, INSERM U1148, LVTS, F-75018, Paris, France; 4 Laboratoire d’Hématologie Biologique, AP-HP, Hôpital Bichat, F-75018, Paris, France; 5 Anesthesiology Department and Surgical Intensive Care, DMU PARABOL, AH-HP, Bichat–Claude Bernard Hospital, Paris, France; 6 Laboratory of Vascular Translational Science, Paris Cité University, INSERM, Paris, France; 7 Intensive Care Medicine, AP-HP, Hôpital Bichat - Claude Bernard, Paris, France

**Keywords:** heparin-induced thrombocytopenia, therapeutic plasma exchange, platelet factor 4, platelet activation, extracorporeal membrane oxygenation

## Abstract

Therapeutic plasma exchange (TPE) has been proposed to remove heparin-induced thrombocytopenia (HIT) antibodies before planned thoracic surgery in patients with acute HIT and to allow brief re-exposure to heparin during surgery. In patients on extracorporeal membrane oxygenation (ECMO), simultaneous administration of TPE and alternative nonheparin anticoagulant therapies is challenging.

We report 2 patients on ECMO with acute HIT who underwent repeated TPE to enable cardiothoracic surgery with the use of heparin. In both cases, serial monitoring of HIT antibody titer and heparin-induced platelet activation assay (HIPA) was performed. The effect of adding exogenous platelet factor 4 (PF4) in the HIPA was also tested.

Negative anti-PF4/H IgG levels were achieved after 5 and 3 TPE sessions, respectively and patients could beneficiate from surgery with brief heparin re-exposure without any thrombotic complication. Negative HIPA results were obtained before negative anti-PF4/H IgG in one patient but remained positive in the other despite very low antibody titers. The addition of PF4 in HIPA led to more contrasted results for the two patients.

Serial HIT screening including immunological and functional assays is necessary to closely monitor TPE in acute HIT patients on ECMO who require surgery. The addition of PF4 in HIPA could help detect clinically relevant platelet-activating antibodies and guide re-exposure to heparin.

Heparin-induced thrombocytopenia (HIT) is a serious adverse effect of heparin therapy caused by an immune reaction mediated by platelet-activating immunoglobulin G (IgG) antibodies against platelet factor 4 and heparin (anti-PF4/H) complex. When HIT is suspected, heparin must be immediately stopped and replaced by an alternative anticoagulant such as danaparoid or argatroban. 1 However, use of these molecules during cardiac surgery is associated with an increased risk of bleeding and/or thrombosis. 2 3 Another strategy is to combine intraoperative unfractionated heparin (UFH) with a potent parenteral antiplatelet agent that prevents HIT-mediated platelet aggregation during cardiopulmonary bypass (CBP). 4 5 6 Therapeutic plasma exchange (TPE) has been proposed to remove HIT antibodies and allow the use of UFH during surgery. 7 8 In patients on extracorporeal membrane oxygenation (ECMO), simultaneous administration of TPE and argatroban is challenging. Only a few case reports are available in the literature with limited biological HIT characterization and follow-up. 9

Here, we report two cases of patients on ECMO with acute HIT who underwent repeated TPE to allow cardiothoracic surgery with the use of UFH. We also evaluated the interest of adding recombinant PF4 in the platelet functional test to increase its performance in HIT monitoring in this context. 10 11

## Case Report

The study was performed in accordance with the Declaration of Helsinki and the institutional review board approved the study. Both patients included were informed of the research protocol by letter, allowing them to express their opposition to the use of their data, according to French legislation and the institutional review board.

HIT antibody testing was performed on citrated plasma samples using a commercial enzyme-immunoassay (EIA) specific for PF4/H IgG (Zymutest HIA IgG, Hyphen BioMed, France). Results were expressed in optical density (OD) units and values >0.5 were considered positive. Plasma samples were then heated at 56°C for 30 minutes to inactivate traces of thrombin and were stored at −80°C for functional test. Heparin-induced platelet activation assay (HIPA) was performed as previously reported. 12 13 In HIPA + PF4 experiments, 10 µg/mL recombinant human PF4 (Hyphen Biomed) was added with low and high concentrations of UFH.

Routine coagulation tests and measurement of argatroban anti-IIa activity (Hemoclot Thrombin Inhibitors, Hyphen Biomed) were performed on an ACL TOP 700 analyzer (Werfen, Spain). Pre- and post-TPE biological monitoring included platelet count, platelet-activating anti-PF4/H antibodies as well as routine coagulation tests and repeated ionized calcium. We used the PRISMAFLEX TPE 2000 set (Baxter, United States) with fresh-frozen plasma (FFP) for TPE sessions in all patients. Vascular access was obtained through a central venous dialysis catheter placed in the jugular vein.

## Patient #1

**Fig. 1:**
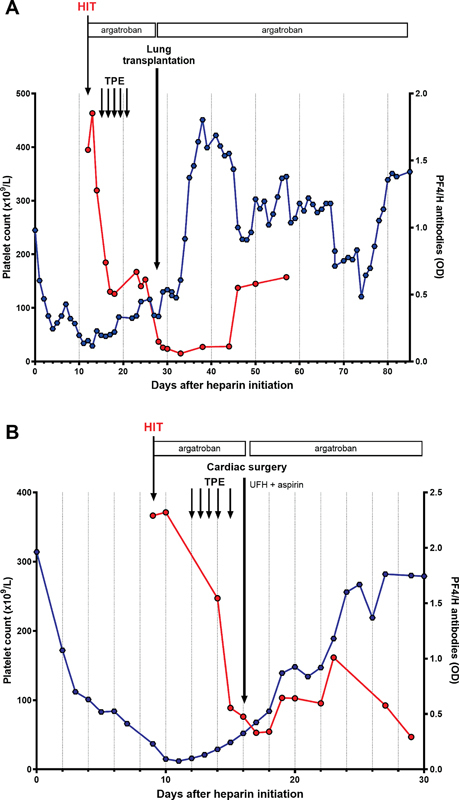
Platelet count and anti-PF4/H IgG level kinetics before and after surgery in patient #1 ( **A** ) and patient #2 ( **B** ). HIT, heparin-induced-thrombocytopenia; OD, optical density; PF4, platelet factor 4; TPE, therapeutic plasma exchange; UFH, unfractionated heparin.

A 36-year-old woman was referred to our lung transplantation center in intensive care unit (ICU) for acute exacerbation of progressive-fibrosing interstitial lung disease. Three days after admission, she was placed on veno-arterial femoro-femoral ECMO (VA-FF-ECMO) support and anticoagulation with UFH was started (Day 1, D1). The primary indication for VA-FF-ECMO as a bridge to lung transplantation was severe hypoxemia and right heart failure. The patient was registered in the nationwide high-priority allocation program according to lung-transplant allocation modalities in France. 14 At D8, she developed recurrent thrombosis of the ECMO circuit despite UFH therapy, concurrently with a drop in platelet count (nadir D13, 29 × 10 ^9^ /L). HIT was suspected and anti-PF4/H IgG were found positive (1.58 OD; Fig. 1A ). Circuit membrane was changed and UFH was immediately stopped. Anticoagulation with argatroban was initiated with a target anti-IIa activity between 0.8 and 1.2 µg/mL. HIT was later confirmed by HIPA ( Table 1 ).

**Table 1:** Evolution of heparin-induced thrombocytopenia laboratory assays following therapeutic plasma exchanges

	Day after heparin initiation	TPE (number)	Anti-PF4/H IgG (OD)	Platelet functional assays	
				HIPA a	HIPA + PF4
Patient #1	12		1.58	≥2 positive	ND
	13	1	1.85	≥2 positive	ND
	14	2	1.28	≥2 positive	ND
	13	3	ND	ND	ND
	16	4	0.74	≥2 positive	ND
	17	5	0.52	≥2 positive	≥2 positive
	18		0.51	1 positive 2 low positive 1 negative	≥2 positive
	24		0.56	≥2 positive	≥2 positive
	**28**		**0.15**	**1 positive** **3 negative**	**1 low positive** **3 negative**
	29		0.14	1 positive 3 negative	4 negative
Patient #2	9		2.29	≥2 positive	≥2 positive
	10		2.32	1 positive	1 positive
	12	1	ND	ND	ND
	14	2	1.55	4 negative	≥2 positive
	15	3	0.56	4 negative	4 negative
	16	4	0.48	4 negative	4 negative
	**17**		**0.48**	**1 low positive** **3 negative**	**4 negative**
	18		0.48	1 low positive 3 negative	4 negative

Abbreviations: Anti-PF4/H, anti-PF4-heparin; HIPA, heparin-induced platelet activation; IgG, immunoglobulin G; ND, not determined; OD, optical density; PF4, platelet factor 4; TPE, therapeutic plasma exchange.

Note: In bold, the day of surgery. Overall interpretation of HIPA or HIPA + PF4: red = positive; orange = low positive; green = negative.

a The sample was considered positive for HIT with HIPA if: (1) the suspension became transparent due to platelet aggregation with UFH 0.2 IU/mL and/or 0.5 IU/mL but not with UFH 48 IU/mL, (2) positive results were obtained with at least two platelet donors within 25 minutes. The sample was considered low positive for HIT if suspension became not completely transparent with UFH 0.2 IU/mL and/or 0.5 IU/mL. The sample was considered negative for HIT if negative results were obtained with four donors. Positive and negative control plasma were run in parallel in each series.

In anticipation of surgery in this HIT patient on VA-FF-ECMO with therapeutic dose of anticoagulation, a multidisciplinary team discussed the intraoperative need of a cell saver involving a bolus of UFH and the associated risk of reaction with HIT antibodies. Given the poor response to first-line desensitization regimen with intravenous immunoglobulin, TPE was decided to remove both anti-HLA and anti-PF4/H antibodies and to allow lung transplantation. Consequently, between D13 and D17, a total of five TPE sessions were performed using 5% albumin during the first two sessions and 50% albumin/FFP for the last three. This approach aimed to minimize hemodilution and decrease in coagulation proteins, particularly important in this high bleeding-risk preoperative setting. Furthermore, unlike albumin, IgG-containing FFP is highly effective in inhibiting HIT antibody-mediated platelet activation. 15 While anti-PF4/H antibodies decreased from 1.85 OD on D13 to 0.50 OD on D18, HIPA and HIPA + PF4 results remained positive. On D28, anti-PF4/H antibodies decreased further (0.15 OD), whereas both HIPA and HIPA + PF4 remained positive with one platelet donor and weakly positive with another one. Lung transplantation was performed while the patient was still on ECMO. Surgery was complicated by a hemorrhagic shock requiring transfusion with 9 red blood cell concentrates, 7 units of FFP, 6 g of fibrinogen and tranexamic acid. Ultimately, intraoperative blood loss was estimated at 4,000 mL and was managed by the intraoperative blood salvage system (Cell Saver System, Fresenius Kabi, Germany) using 25,000 IU of UFH for anticoagulation of the system. Anticoagulation with argatroban was restarted 6 hours after surgery. On the day after surgery (D29), anti-PF4/H remained low (0.13 OD) but HIPA was still positive with one platelet donor while HIPA + PF4 was negative. Patient was successfully weaned from ECMO support. Platelet count increased from 69 × 10 ^9^ /L on D28 to 451 × 10 ^9^ /L on D38, 10 days after surgery. During follow-up, no thrombotic or bleeding event occurred and platelet count remained normal. The patient was discharged from ICU at D122. One year after lung transplantation, she died of chronic allograft dysfunction and severe infection.

## Patient #2

A 63-year-old patient was admitted for cardiogenic shock and multiple organ failure requiring emergent VA-FF-ECMO implantation and anticoagulation with UFH at therapeutic dose (D1). His medical history included an acute coronary syndrome treated with coronary stenting 1 month before. At this time point, a treatment with dual antiplatelet therapy using aspirin and clopidogrel, and rivaroxaban 20 mg per day was started. After VA-FF-ECMO implantation, the patient was found to have a postinfarction ventricular septal defect (PIVSD). A delayed surgical repair was decided in order to improve his hemodynamic parameters and to increase the success rate of PIVSD surgery. He developed progressive thrombocytopenia from 383 × 10 ^9^ /L on D1 to 66 × 10 ^9^ /L on D8 that was attributed to ECMO. On D9, a further fall in platelet count to 37 × 10 ^9^ /L was associated to thrombus deposition in the ECMO circuit and the oxygenator. Circuit membrane was changed, then HIT was suspected, UFH was immediately stopped and argatroban was initiated. Anti-PF4/H antibodies were strongly positive (2.29 OD) and HIT was further confirmed by HIPA ( Fig. 1B ). Surgical treatment of interventricular communication led to four TPE sessions with 100% FFP from D12 to D16. We performed TPE sessions using 100% FFP in this patient in the pre-CBP setting, due to a high risk of bleeding. Our aim was to minimize bleeding complications due to hemodilution and the decrease in coagulation proteins, particularly fibrinogen, associated with TPE. 2 Consequently, anti-PF4/H antibodies progressively decreased (1.55 OD and 0.56 OD after the first and second TPE sessions, respectively) and became negative after the third TPE (0.48 OD on D16). By the time the results for anti-PF4/H antibodies were obtained after the third TPE, a fourth TPE had already been performed. Interestingly, HIPA became negative from the first TPE, whereas HIPA + PF4 became negative only after the second TPE. Patient underwent cardiac surgery with CBP on D17 with UFH 20,000 IU and aspirin 300 mg without thrombotic complication. He received 6 units of FFP, 3 red blood cell concentrates, and 1 platelet concentrate because of excessive bleeding. Argatroban was reintroduced postoperatively. ECMO support was successfully withdrawn the day after cardiac surgery (D18). Platelet count normalized at D20. After surgery, anti-PF4/H IgG increased until D23 (1.01 OD) and became negative again at D29 (0.29 OD). HIPA + PF4 remained negative until D22. Unfortunately, the patient died 3 weeks later because of sepsis and multiple-organ failure.

## Discussion

Repeated TPE has been recommended by the American Society for Apheresis to remove HIT antibodies before planned cardiac/vascular surgery in patients with serologically confirmed acute HIT. 8 In this setting, a negative platelet activation assay such as HIPA has been recommended as the target serological endpoint to allow safe surgery, as EIA could remain strongly positive. 7 In 2018, the American Society of Hematology 1 and in 2023, the British Society for Haematology 16 recommended the use of TPE for patients with HIT who need heparin re-exposure prior to CBP or vascular surgery. This is particularly when heparin use is essential during the surgery, followed by a switch to a nonheparin anticoagulant post-surgery. However, there is no consensus regarding the composition of TPE and FFP. Additionally, in 2019, French guidelines recommended combining intraoperative anticoagulation with UFH and an intravenous antiplatelet agent (either tirofiban or cangrelor). 17

Here, we report two patients on ECMO with acute HIT who beneficiated from TPE before thoracic surgery. TPE sessions are isovolemic, resulting in a globally neutral fluid balance postprocedure. Although patients underwent hemodynamic monitoring (invasive arterial pressure, echocardiography) during their ICU stay, the TPE procedures were remarkably well tolerated by both patients, without any complications, and were not associated with increased fluid requirements.

In both patients, the complete kinetics of anti-PF4/H antibodies were monitored by EIA and HIPA pre- and postoperatively. Negative anti-PF4/H IgG levels were achieved after 5 and 3 TPE sessions in patients #1 and #2, respectively. In patient #1, HIPA remained positive while antibody titer was very low (0.12 OD). The patient received UFH from the cell saver system. This unexpected kinetics differed from the one previously described, in which negative functional assay was achieved more quickly. 7 It could be explained by the presence of heparin-dependent platelet-activating antibodies different from anti-PF4/H IgG, as the patient had alloantibodies. Besides, in patient #2, HIPA became negative before anti-PF4/H IgG, while HIPA + PF4 became negative after one supplementary TPE session. Thus, addition of PF4 to HIPA allowed detecting platelet-activating antibodies after the first TPE, while standard HIPA was already negative. As the HIPA and HIPA + PF4 were performed at the same time under similar technical conditions and using the same platelet donors, it is likely that these discordances represent clinically relevant differences. Once negative EIA and HIPA + PF4 achieved, patient #2 could beneficiate from CBP with high doses of UFH without any thrombotic complication.

These two cases suggest that TPE could be a safe and effective peri-operative strategy to remove platelet-activating anti-PF4/H antibodies. Moreover, this strategy allows brief re-exposure to heparin during CBP in the acute phase of HIT without thrombotic complications associated with the use of alternative anticoagulant therapies. Finally, our findings emphasize the need to perform serial and complete HIT screening including immunological and functional assays during TPE and suggest that the addition of PF4 in HIPA is more discriminant to detect platelet-activating antibodies and help deciding when patient is suitable for heparin re-exposure. Our results also suggest that the achievement of a negative EIA could be as useful as a negative HIPA + PF4 for brief surgical UFH re-exposure. Since many laboratories have not validated HIT platelet functional assays, in particular with addition of PF4, the confirmation of these results in a further and larger multicenter study is of major importance to guide and homogenize practices.
